# Accurate Truncations of Chain Mapping Models for Open Quantum Systems

**DOI:** 10.3390/nano11082104

**Published:** 2021-08-19

**Authors:** Mónica Sánchez-Barquilla, Johannes Feist

**Affiliations:** Departamento de Física Teórica de la Materia Condensada and Condensed Matter Physics Center (IFIMAC), Universidad Autónoma de Madrid, E-28049 Madrid, Spain; monica.sanchezb@uam.es

**Keywords:** nanophotonics, quantum dissipation, chain mapping, non-Markovian dynamics, spectral density, harmonic oscillators

## Abstract

The dynamics of open quantum systems are of great interest in many research fields, such as for the interaction of a quantum emitter with the electromagnetic modes of a nanophotonic structure. A powerful approach for treating such setups in the non-Markovian limit is given by the chain mapping where an arbitrary environment can be transformed to a chain of modes with only nearest-neighbor coupling. However, when long propagation times are desired, the required long chain lengths limit the utility of this approach. We study various approaches for truncating the chains at manageable lengths while still preserving an accurate description of the dynamics. We achieve this by introducing losses to the chain modes in such a way that the effective environment acting on the system remains unchanged, using a number of different strategies. Furthermore, we demonstrate that extending the chain mapping to allow next-nearest neighbor coupling permits the reproduction of an arbitrary environment, and adding longer-range interactions does not further increase the effective number of degrees of freedom in the environment.

## 1. Introduction

No quantum system is ever fully isolated. Instead, any system is coupled to its external environment with a large (essentially infinite) number of degrees of freedom, leading to the notion of open quantum systems [1,2,3]. When considering a specific setup, the distinction between which part of the whole is considered the “system” and which the “environment” (or “reservoir” or “bath”) is in principle arbitrary, but is often based on a clear distinction of the physical properties of the system and the environment. Some typical environments are, for example, formed by electromagnetic modes [4], phonons [5], or ensembles of other quantum systems [6]. When perturbations of the environment caused by the system do not act back on the system at later times, the environment essentially has no “memory” about the dynamics that the system underwent at earlier times. In this so-called Markovian limit, the dynamics of the system can be approximately described using a Lindblad master equation [7]. We note that lack of memory does not necessarily require or imply that the environment fully equilibrates to its original state—for example, a photon emitted from an atom in free space will never interact with the atom again even if it is not absorbed or otherwise affected by another object after being emitted.

**Figure 1:**
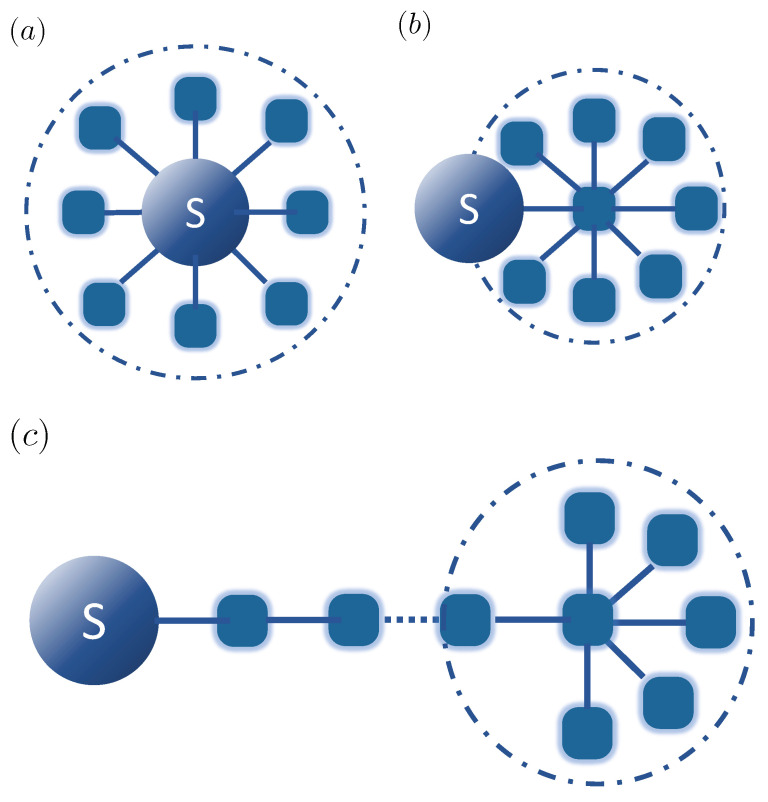
(**a**) Quantum system coupled to a discrete set of environment modes; (**b**) quantum system coupled to a reaction mode (a collective environment mode), with this mode coupled to a residual bath of modes; (**c**) chain mapping for the environment modes after *n* steps, with a residual bath of N−n modes at the end of the chain.

While the Markovian approximation is extremely useful in many situations, there are many cases in which it does not apply and which thus require a more general treatment. For concreteness, we assume that the environment is well-approximated as a collection of harmonic oscillators, and that all relevant environment modes are of the same nature and thus interact with the system through the same system operator $A_{S}$. The Hamiltonian that describes a system *S* interacting with a harmonic environment, schematically depicted in Figure 1a, can then be written as (with ℏ=1)

(1) $$H_{0}=H_{S}+\sum_{k}\frac{\omega_{k}}{2}\left(\frac{p_{k}^{2}}{\alpha_{k}^{2}}+\alpha_{k}^{2}x_{k}^{2}\right)+A_{S}\sum_{k}c_{k}\alpha_{k}x_{k},$$

where $H_{S}$ is the bare system Hamiltonian, $A_{S}$ is the system part of the system–bath interaction operator, and $x_{k}=\frac{1}{\alpha_{k}\sqrt{2}}\left(a_{k}^{†}+a_{k}\right)$ and $p_{k}=\frac{i\alpha_{k}}{\sqrt{2}}\left(a_{k}^{†}-a_{k}\right)$ are (generalized) position and momentum operators for the *k*th harmonic oscillator with frequency $\omega_{k}$, with an arbitrary scaling factor $\alpha_{k}$ that preserves the commutation relation $\left[x_{k},p_{l}\right]=i\delta_{kl}$. We note that $\alpha_{k}x_{k}$ and $p_{k}/\alpha_{k}$ are unitless, such that the units of $x_{k}$ and $p_{k}$ are determined by $\alpha_{k}$. For example, the choice $\alpha_{k}=\sqrt{m\omega_{k}}$ gives the normal position and momentum operators for a massive particle with mass *m*. The real constants $c_{k}$ characterize the coupling between the bath modes and the system, with units of frequency if $A_{S}$ is chosen as unitless. The general form given in Equation (1) can represent any physical system that can be (approximately) described by a collection of harmonic oscillators, such as the photonic modes of a (nano)cavity [8] or the rovibrational modes of a molecule and solvent.

The information about the bath and its coupling to the system is fully encoded in the so-called spectral density $J_{0}\left(\omega \right)=\sum_{k}c_{k}^{2}\delta \left(\omega -\omega_{k}\right)$. In many cases of practical importance, the bath modes are either continuous in frequency or their density is high enough that $J_{0}\left(\omega \right)$ can be treated as a continuous function. For example, when describing the interaction between a two-level emitter and the electromagnetic modes of an arbitrary environment described through macroscopic electromagnetism, the spectral density is given by

(2) J0(ω)=ω2μe2πϵ0c2n→e·Im{G(r→e,r→e,ω)}·n→e,

where $\mu_{e}$ is the transition dipole moment of the emitter, ${\vec{n}}_{e}$ is its orientation, ${\vec{r}}_{e}$ is its position, and $G({\vec{r}}^{\;\prime },\vec{r},\omega )$ is the dyadic Green’s function of the electromagnetic environment between points $\vec{r}$ and ${\vec{r}}^{\;\prime }$ [8,9,10,11]. In general, structured spectral densities indicate that the environment has memory, i.e., its influence on the system depends on interactions at earlier times, while featureless spectral densities indicate memoryless (Markovian) environments [1,2,3].

Over the last few decades, many methods to describe non-Markovian dynamics in arbitrary environments have been developed, among them the hierarchical equations of motion (HEOM) [12,13,14,15], stochastic methods [16,17,18], and time-adaptive density matrix renormalization group treatments [19,20,21]. In general, these descriptions are computationally costly, especially if the dynamics at long times are of interest.

In this context, a general conceptual approach is to transform structured baths in such a way that they either become amenable to approximate treatments or take a form for which specific numerical treatments become more efficient due to the transformed structure of the problem. In particular, such approaches often are based on the general idea of enlarging the system by including one (or several) “reaction modes” of the environment within it in such a way that these modes contain the “memory” of the bath, and are then in turn coupled to a new residual bath that ideally has little or no memory. Among these approaches are the pseudomode method [22,23,24,25], effective Lindblad master equation procedure [26], and reaction coordinate mappings [27,28,29,30]. In such a mapping, an orthogonal transformation is applied on the bath modes such that the interaction between the system and the full environment is captured by a single mode, which itself interacts with the residual bath (Figure 1b). Applying this approach iteratively leads to a chain-like description of the environment (Figure 1c), in which each mode is only coupled with its nearest neighbors [20,31,32,33]. In particular, this representation allows a numerically efficient treatment based on tensor networks/matrix product states [34,35]. Still, this technique is limited by the scaling of the computational effort with the size of the system, as long chains need to be used to describe the dynamics up to long times, and correlations in the system grow over time, leading to an overall unfavorable scaling with propagation time. In some cases, this can be mitigated by using the transfer tensor method, which makes the scaling linear with propagation time [36,37].

In this work, we first review the reaction coordinate mapping and its extension to the chain mapping. We show that the so-called “particle” and “phonon” mappings, which in the literature have been derived based on a parameterized Bogoliubov transformation [33], are natural consequences of performing the mapping with differently scaled parameters (i.e., different choices of $\alpha_{k}$ in Equation (1)). We then explore different methods to obtain an accurate description of the system dynamics when the chain is truncated to some maximum length $N_{C}$. We first take inspiration from the well-known complex absorbing potentials used in quantum mechanics to smoothly absorb wavepackets emitted into the chain without reflections. We then generalize this approach to a fitting procedure inspired by Ref. [38] where the chain parameters are optimized to obtain an accurate description of the system dynamics with even shorter chains, and finally show that this description can be significantly improved by allowing up to next-nearest neighbor couplings between the chain sites. Somewhat surprisingly, we find indications that, for a given number of sites $N_{C}$, a chain with next-nearest neighbor coupling already allows for finding the optimal description, and adding longer-range interactions does not further increase the quality of the fit.

## 2. Materials and Methods

We here provide an overview of the reaction coordinate mapping and related chain transformation for a reservoir or bath of harmonic oscillators interacting with a physical system. While there are several excellent reviews in the literature, most focus on only one specific mapping. On the other hand, while Ref. [33] introduces a parameterized formulation that generalizes and contains previous approaches, it focuses on the mathematical aspects of such approaches. We thus focus on a short and intuitive exposition here. In particular, we show that the so-called “phonon” and “particle” mappings, which arise in Ref. [33] as special cases of a parametrized Bogoliubov transformation, can also be understood as coordinate transformations in differently scaled coordinates for the harmonic oscillators.

In the following, we assume that the spectral density is nonzero only in the interval $\left[\omega_{L},\omega_{R}\right]$. The upper limit $\omega_{R}$ is assumed to be finite, either because of physical constraints or because of the introduction of an artificial cutoff (with higher frequencies assumed to only contribute a renormalization of the system frequencies). The lower limit $\omega_{L}$ is equal to zero in many cases of practical interest. However, a special case is given by baths at finite temperatures, which can be replaced by an effective bath at zero temperature with an extended frequency range starting at $\omega_{L}=-\omega_{R}$ [39]. In the following, we thus assume that the bath is at zero temperature without any loss of generality.

The reaction coordinate transformation is an orthogonal coordinate transformation, $\vec{X}=O\vec{x}$ and $\vec{P}=O\vec{p}$, where *O* is an orthogonal matrix, $OO^{T}=O^{T}O=1$, and the first basis vector (the reaction coordinate) is chosen so that it contains the full coupling between the system and the bath:

(3) $$X_{1}=\sum_{k}\frac{c_{k}\alpha_{k}x_{k}}{D_{1}}=\sum_{k}O_{1k}x_{k},$$

where $D_{1}$ is a normalization constant that ensures $\sum_{k}O_{1k}^{2}=1$, i.e., $D_{1}^{2}=\sum_{k}c_{k}^{2}\alpha_{k}^{2}$. The Hamiltonian after the transformation is

(4) $$H_{1}=H_{S}+D_{1}A_{S}X_{1}+\sum_{k,i,j}\frac{\omega_{k}}{2}O_{ik}O_{jk}\left(\frac{P_{i}P_{j}}{\alpha_{k}^{2}}+\alpha_{k}^{2}X_{i}X_{j}\right).$$

In the following, we show that the choices $\alpha_{k}={\tilde{\alpha }}_{k}=\sqrt{\omega_{k}}$ and $\alpha_{k}=1$ are equivalent to the phonon mapping and particle mapping of Ref. [33], respectively. For ${\tilde{\alpha }}_{k}=\sqrt{\omega_{k}}$, which corresponds to mass-scaled coordinates in which the kinetic term has the same form for all oscillators, the transformed Hamiltonian is

(5) $${\tilde{H}}_{1}=H_{S}+{\tilde{D}}_{1}A_{S}{\tilde{X}}_{1}+\frac{1}{2}\left(\sum_{i}{\tilde{P}}_{i}^{2}+\sum_{ij}{\tilde{C}}_{ij}{\tilde{X}}_{i}{\tilde{X}}_{j}\right).$$

In this scaling, the momentum operators are diagonal, but the potential energy includes linear couplings between the oscillator coordinates, given by ${\tilde{C}}_{ij}=\sum_{k}\omega_{k}^{2}O_{ik}O_{jk}$. The diagonal elements ${\tilde{C}}_{ii}$ determine the frequencies of the transformed bath modes. In particular, the reaction coordinate frequency follows from the definition of $X_{1}$ as ${\tilde{\Omega }}_{1}^{2}={\tilde{C}}_{11}=\sum_{k}c_{k}^{2}\omega_{k}^{3}/{\tilde{D}}_{1}^{2}=\frac{\sum_{k}c_{k}^{2}\omega_{k}^{3}}{\sum_{k}c_{k}^{2}\omega_{k}}$.

Apart from $O_{1k}$, the transformation is not specified. This allows significant liberty to choose the form of the coupling matrix ${\tilde{C}}_{ij}$. In particular, it can be chosen to be tridiagonal (and explicitly constructed as such by using, e.g., the Lanczos algorithm), which directly describes the bath as a chain of harmonic oscillators coupled through nearest-neighbor interactions [33]. For sufficiently well-behaved (in particular, gapless) spectral densities, at each step in the chain, the spectral density of the “residual” bath, i.e., the rest of the chain, can be obtained analytically from that of the previous bath [28,29,33,40], and is given by

(6) $${\tilde{J}}_{n+1}\left(\omega \right)=\frac{{\tilde{D}}_{n+1}^{2}{\tilde{J}}_{n}\left(\omega \right)}{{\left|{\tilde{W}}_{n}^{+}\left(\omega \right)\right|}^{2}},$$

where ${\tilde{W}}_{n}^{+}\left(\omega \right)$ is given by

(7) $${\tilde{W}}_{n}^{+}\left(\omega \right)=\lim_{\epsilon \to 0^{+}}\int_{0}^{\infty }\frac{\nu {\tilde{J}}_{n}\left(\nu \right)}{\nu^{2}-{\left(\omega +i\epsilon \right)}^{2}}d\nu .$$

For sufficiently long chains (n→∞), the spectral density in this mapping converges to

(8) $${\tilde{J}}_{\infty }\left(\omega \right)=\frac{\sqrt{\left(\omega^{2}-\omega_{L}^{2}\right)\left(\omega_{R}^{2}-\omega^{2}\right)}}{\pi },$$

which is independent of any properties of the original spectral density apart from its frequency support. In this limit, the mode frequency and coupling also tend to limiting values, ${\tilde{\Omega }}_{\infty }^{2}=\frac{\omega_{R}^{2}+\omega_{L}^{2}}{2}$ and ${\tilde{D}}_{\infty }=\frac{\omega_{R}^{2}-\omega_{L}^{2}}{4}$.

If the reaction coordinate mapping is instead done using $\alpha_{k}=1$, which corresponds to “natural” coordinates in which *x* and *p* are unitless and appear symmetrically in the Hamiltonian, the transformed Hamiltonian is

(9) $$H_{1}=H_{S}+D_{1}A_{S}X_{1}+\frac{1}{2}\sum_{i,j}C_{ij}\left(P_{i}P_{j}+X_{i}X_{j}\right),$$

with $C_{ij}=\sum_{k}\omega_{k}O_{ik}O_{jk}$. The diagonal elements again determine the new mode frequencies, now with $\Omega_{i}=C_{ii}$, with the reaction coordinate frequency in this mapping given by $\Omega_{1}=\frac{\sum_{k}c_{k}^{2}\omega_{k}}{\sum_{k}c_{k}^{2}}$. Rewriting Equation (9) in terms of ladder operators $A_{k}=\frac{1}{\sqrt{2}}\left(X_{k}+iP_{k}\right)$, and again choosing $C_{ij}$ to be tridiagonal leads to a chain Hamiltonian of the form

(10) $$H_{c}=H_{S}+\lambda_{1}A_{S}\left(A_{1}+A_{1}^{†}\right)+\sum_{i}\Omega_{i}A_{i}^{†}A_{i}+\sum_{i}\lambda_{i+1}\left(A_{i}A_{i+1}^{†}+A_{i}^{†}A_{i+1}\right),$$

where $\lambda_{1}=\frac{1}{\sqrt{2}}D_{1}$, and we have dropped a constant contribution $\sum_{i}\frac{1}{2}\Omega_{i}$. In this form, it is seen immediately that the coupling between the different chain sites conserves the number of excitations (quasiparticles), such that this corresponds to the “particle mapping” as defined in Ref. [33]. We stress that the absence of counterrotating terms that do not conserve the excitation number is *not* due to a rotating wave or similar approximation but is exact and directly follows from the choice of coordinate scaling.

In the particle mapping, the recursion relation for the residual spectral density after *n* steps is given by

(11) $$J_{n+1}\left(\omega \right)=\frac{\lambda_{n+1}^{2}J_{n}\left(\omega \right)}{\left|W_{n}^{+}\left(\omega \right)\right|},$$

where

(12) $$W_{n}^{+}\left(\omega \right)=\lim_{\epsilon \to 0^{+}}\int_{0}^{\infty }\frac{J_{n}\left(\nu \right)}{\nu -\left(\omega +i\epsilon \right)}d\nu ,$$

with the limiting spectral density for n→∞ being

(13) $$J_{\infty }\left(\omega \right)=\frac{\sqrt{\left(\omega -\omega_{L}\right)\left(\omega_{R}-\omega \right)}}{2\pi },$$

with limiting values $\Omega_{\infty }=\frac{\omega_{L}+\omega_{R}}{2}$ and $\lambda_{\infty }=\frac{\omega_{R}-\omega_{L}}{4}$.

## 3. Results

**Figure 2:**
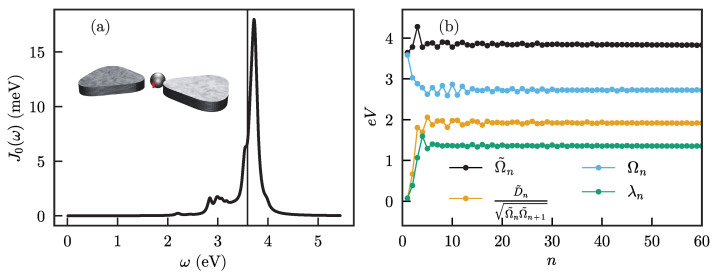
(**a**) Spectral density for an emitter in the combined sphere-bowtie antenna (illustrated in the inset). The emitter is situated at the center of the bowtie (indicated by the red arrow), with its frequency chosen close to the maximum of the spectral density (indicated by the thin vertical line); (**b**) chain mapping frequencies ${\tilde{\Omega }}_{n}$, $\Omega_{n}$ and coupling parameters ${\tilde{D}}_{n}$, $\lambda_{n}$ for the phonon and particle mapping.

The system we consider is a two-level emitter with transition frequency $\omega_{e}$ and transition dipole moment $\mu_{e}$ placed in the central gap of a nanoantenna consisting of a bowtie coupled to a nanosphere, as shown in the inset of Figure 2a. For this case, the system operators are $H_{S}=\omega_{e}\sigma^{+}\sigma^{-}$ and $A_{S}=\sigma^{+}+\sigma^{-}$, where $\sigma^{+}$ and $\sigma^{-}$ are the two-level raising and lowering operators, respectively. Note that the transition dipole moment $\mu_{e}$ is included in the definition of the spectral density and $A_{S}$ is unitless. The spectral density, given by Equation (2), is shown in Figure 2a and is calculated using the SCUFF-EM package [41,42].

The results of the phonon and particle chain mappings for this spectral density are shown in Figure 2b. On the first sites of the chain, the frequencies and couplings vary strongly, but then quickly converge to their final values and stay constant. Sufficiently far from the system, the infinitely long tail of the chain thus becomes translationally invariant. The chain mapping thus provides a description that closely resembles a spatial discretization of one-dimensional motion in a short-range “potential” that disappears at a sufficient distance from the system. This implies that any excitation that propagates along the chain will not be reflected anymore after reaching a sufficient distance, and can thus not affect the system. This general structure implies that the chain mapping naturally provides a separation of any bath into (i) a non-Markovian part close to the system, where excitations can be reflected due to the position-dependent coupling and energy at each site, and then interact again with the system, leading to memory effects, and (ii) a Markovian part far enough away from the system where excitations propagate along a featureless continuum, such that their ‘momentum’ along the chain is conserved and they are just transported away without ever affecting the system again. However, there is no sharp boundary between the two parts, and the transition is gradual.

**Figure 3:**
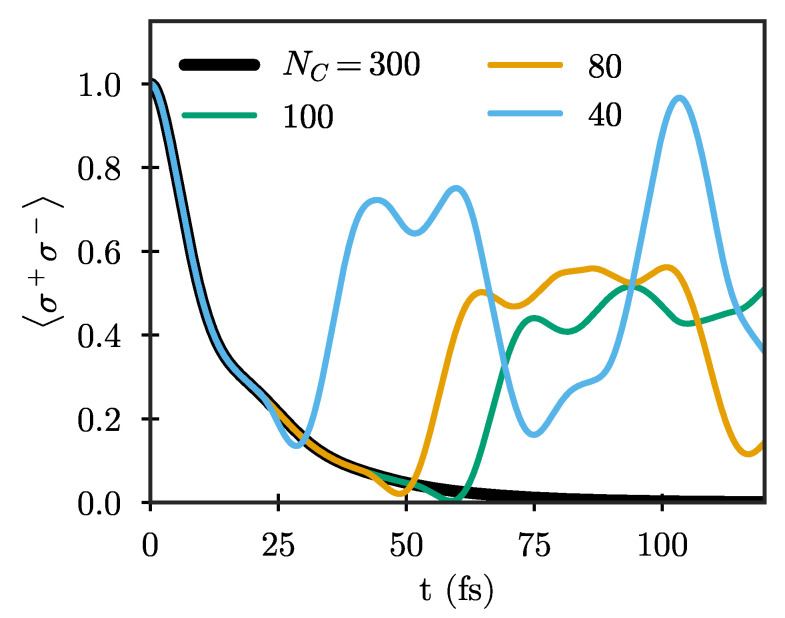
Excited-state population of an initially excited two-level quantum emitter with frequency $\omega_{e}=3.6$ eV and dipole moment $\mu_{e}=0.6$ e nm coupled to the antenna shown in Figure 2a, calculated using chains truncated after different numbers of sites $N_{C}$.

In practical calculations, it is not possible to explicitly treat an infinite chain, and the chain has to be truncated at some maximum length. When this truncation is performed by simply removing all chain sites after a given distance from the system, the wave packets propagating along the chain are reflected back and will interact with the system again after some time. Such an approach thus limits the numerical simulations to propagation times smaller than roughly twice the propagation time along the chain [35,37,43,44]. This is demonstrated in Figure 3, which shows the Wigner–Weisskopf dynamics of a quantum emitter with frequency $\omega_{e}=3.6$ eV for different chain lengths. Here, and in the following, we show results only for the particle mapping in which the number of excitations in the chain is a conserved quantity, which is advantageous for quantum simulations. The shortest chain shown ($N_{C}=40$) is already long enough so that both $\Omega_{n}$ and $\lambda_{n}$ have essentially reached their limiting values. Still, the emitter dynamics are clearly not well described for t⪆25 fs due to unphysical reflections from the end, with the maximum time for which the dynamics are well-described increasing as the chain length increases. The results using $N_{C}=300$ are exact for the whole range of times considered here (up to T=120 fs), and will be taken as the reference result in the following.

**Figure 4:**
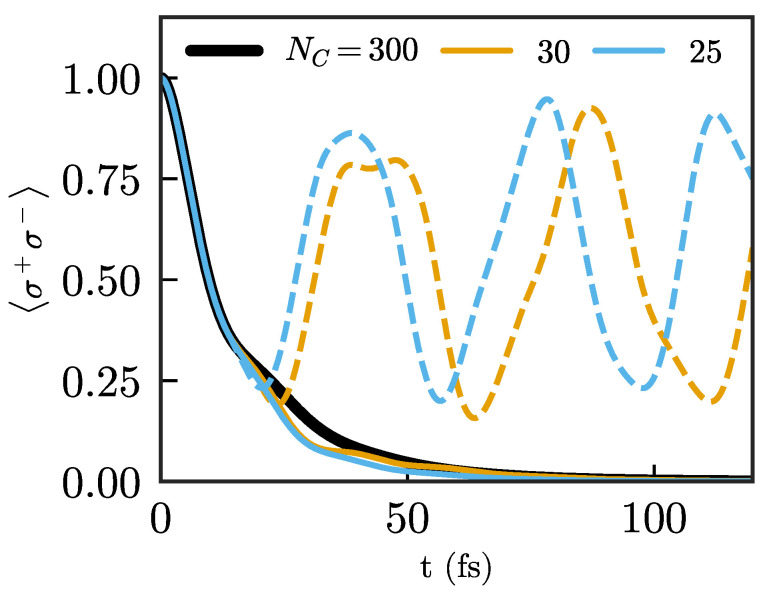
Population of the same emitter as in Figure 3 for two different chain lengths: $N_{C}=30$ (orange) and $N_{C}=25$ (blue) compared with a long chain ($N_{C}=300$), which is considered exact. Comparison between the chains without any absorbing terms (dashed lines) and chains with a single absorbing term at their end, given by $\gamma =2\pi J_{N_{C}}\left(\Omega_{N_{C}}\right)$.

The truncated chains have to become very long to be able to correctly describe the system dynamics at large times. However, the emitted wave packets cannot influence the system anymore after having propagated far enough along the chain, and it thus should not actually be necessary to treat them explicitly at larger distances. This simple fact motivates the central question of this study: How can the chain mapping be truncated such that a finite chain fully reproduces the dynamics of the infinite system? Since the residual spectral density describing the rest of the chain far enough away from the system is relatively smooth (see Equations (8) and (13)), the most straightforward approach is to simply treat the rest as a Markovian bath acting on the last chain site. This approximation replaces all chain sites with $n>N_{C}$ by a Lindblad term in the master equation for the density matrix ρ describing the system and chain up to site $n=N_{C}$:

(14) $$\partial_{t}\rho =-i\left[H,\rho \right]+\gamma L_{A_{N_{C}}}\left[\rho \right],$$

where $\gamma =2\pi J_{N_{C}}\left(\Omega_{N_{C}}\right)$ and $L_{O}\left[\rho \right]=O\rho O^{†}-\frac{1}{2}\left\{O^{†}O,\rho \right\}$. We also note that, in the particle mapping that we focus on here, and assuming that $N_{C}$ is chosen large enough so the limiting residual spectral density is obtained, the residual spectral density is symmetric about $\Omega_{N_{C}}$, and there is no Lamb shift (i.e., bath-induced energy shift) on the mode frequency. The emitter dynamics using this approximation, shown in Figure 4, are much more similar to the exact results compared to the abrupt truncation for the same chain length. However, they clearly do not provide perfect agreement, i.e., they still suffer from residual reflections. This is not too surprising, as the residual spectral densities even far away from the system, given by Equations (8) and (13), are structured and not actually Markovian (which would correspond to a constant spectral density spanning an infinite frequency range). This implies that a “naive” Markovian approximation that just adds losses on the last chain site will necessarily lead to reflections.

We thus take inspiration from the fact that the chain resembles a discretized continuum, i.e., the goal of minimizing reflections on the chain is similar to the goal of absorbing outgoing waves in numerical simulations of continuous systems. This suggests that smoothly increasing losses along the chain, similar to the complex absorbing potentials (CAPs) used in quantum mechanics [45,46,47], could significantly suppress reflections along the chain. The corresponding master equation then becomes

(15) $$\partial_{t}\rho =-i\left[H,\rho \right]+\sum_{n}\gamma_{n}L_{A_{n}}\left[\rho \right],$$

where the $\gamma_{n}$ has to be chosen so as not to affect the dynamics close to the system, avoid reflections due to abrupt changes in the decay rate, and simultaneously achieve absorption of all outgoing wavepackets before reaching the end of the (truncated) chain.

**Figure 5:**
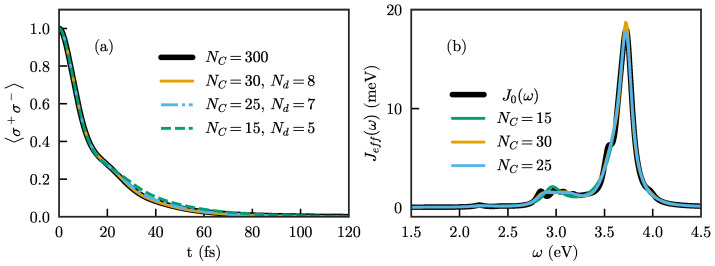
(**a**) Population for the quantum emitter for three different chain lengths: $N_{C}=30$ (orange), $N_{C}=25$ (blue) and $N_{C}=15$ (green) compared to the “exact” one (black). An absorbing function has been added to the first three chains. The parameters are chosen to give a good description of the dynamics, but a wide range is valid for the first to lengths. When the chain is very short, as for $N_{C}=15$, the prediction of the dynamics starts to break down; (**b**) effective spectral densities when the absorbing terms are added in the Hamiltonian for the same lengths as in (**a**). For $N_{C}=30$ and $N_{C}=25$, the effective spectral density is similar to $J_{0}\left(\omega \right)$, although neither of them describes it in detail. For $N_{C}=15$, the effective spectral density presents a shift in the frequency of the main peak of $J_{0}\left(\omega \right)$, affecting the dynamics.

In order to parametrize the decay rates $\gamma_{n}$, we use a functional form inspired by standard CAPs,

(16) $$\gamma_{n}=\Theta \left(x_{n}\right)\left(\alpha x_{n}+\beta x_{n}^{2}\right),$$

where $x_{n}=\frac{n-N_{d}}{N_{C}-N_{d}}$, Θ(x) is the Heaviside theta function, $N_{d}$ determines the chain site at which losses start, and α and β are real numbers that determine the strength of the absorption. To ensure a good description of the dynamics, $N_{d}$ must be chosen large enough (roughly such that the chain mode frequencies and coupling strengths have reached their asymptotic limits). In Figure 5a, we show the population dynamics of an initially excited quantum emitter with the same frequency as before, $\omega_{e}=3.6$ eV, and different chains with smoothly increasing absorption. In particular, we here choose α=0.02 and β=0.1 and show results for the combinations $\left(N_{C},N_{d}\right)=\left(30,8\right),\left(25,7\right),\left(15,5\right)$.

With this choice of parameters, the emitter dynamics are well reproduced for long enough chains, and no reflections of the population are observed. We note that, while we here show dynamics within the single-excitation subspace (i.e., for the Wigner–Weisskopf problem), it is known that, if the effective spectral density of the chain with losses (which can be obtained with, e.g., the approach of Ref. [38]) agrees with the full spectral density, the results will be identical regardless of the number of excitations. The effective spectral density for the lossy chains used here is shown in Figure 5b, and can be seen to indeed agree quite well with the exact spectral density for the longer chains.

However, while the approach of treating the chain index like a ‘spatial’ coordinate and adding an absorbing potential at the end indeed works, it requires relatively long chains to produce good results. For shorter chains, the requirement that the chain is approximately translation-invariant by the time the absorption sets in cannot be satisfied, and absorption necessarily leads to loss of information about the dynamics. This is, e.g., the case for $N_{C}=15$, for which both the emitter dynamics and the effective spectral density are seen to not agree as well with the exact results as for longer chains even after optimizing the parameters.

### 3.1. Fitted Chain

Then, the question arises whether an optimization process can give an improved description of the open quantum system for a given chain length and if this length can be smaller than minimum length found when manually imposing a smooth “absorption potential”. This approach is inspired by related approaches to replace an arbitrary bath by a system of auxiliary oscillators with losses [24,38], where we here restrict the auxiliary modes to form a chain, i.e., we only allow nearest-neighbor interactions between the modes, with only the first one coupled to the emitter. However, instead of using the analytic chain transformation to obtain the parameters of this new chain, we fit the chain parameters to optimally reproduce the actual bath.

There are two equivalent quantities that fully characterize the bath, which are the spectral density J(ω) and the correlation function C(t), which is essentially the Fourier transform of the spectral density

(17) $$C\left(t\right)=\theta \left(t\right)\int_{-\infty }^{\infty }J\left(\omega \right)e^{-i\omega t}d\omega .$$

As mentioned above, we here assume zero temperature, as a zero-temperature chain can represent any finite-temperature bath by adjusting the chain spectral density to reproduce the (temperature-dependent) power spectral density of the bath [39]. The correlation function $C(t-t^{\prime })$ encodes the action of the bath on the system at time *t* due to the system dynamics at time $t^{\prime }$. This suggests that fitting C(t) up to some maximum time could be a promising strategy when only short-time dynamics are of interest, since one explicitly discards the information about long-time dynamics that is implicitly encoded in J(ω). In order to test this strategy, we fit either the spectral density J(ω) or the correlation function C(t) up to the maximum propagation time.

For the model used here, the effective spectral density of the chain is given by [38]

(18) Jmod(ω)=λ12πIm1H˜−ω11,

where $\tilde{H}$ is a tridiagonal complex symmetric (not Hermitian) matrix, with the tridiagonal real part containing the coupling matrix of the chain, and the diagonal imaginary part encoding the losses, i.e., ${\tilde{H}}_{i-1,i}={\tilde{H}}_{i,i-1}=\lambda_{i}$ and ${\tilde{H}}_{i,i}=\Omega_{i}-\frac{i}{2}\gamma_{i}$. By diagonalizing $\tilde{H}=V\tilde{\Omega }V^{T}$, where *V* is a complex orthogonal (not unitary) matrix containing the eigenvectors, with $V^{T}V=1$, and $\tilde{\Omega }$ is a diagonal matrix containing the (complex) eigenvalues ${\tilde{\omega }}_{i}$, both the spectral density and the correlation function can be written as a simple sum of independent terms,

(19) Jmod(ω)=λ12π∑iImV1i2ωi˜−ω

(20) $$\begin{array}{cc} C_{\mathrm{mod}}\left(t\right) & =\theta \left(t\right)\frac{\lambda_{1}^{2}}{\pi }\sum_{i}V_{1i}^{2}e^{-i{\tilde{\omega }}_{i}t} \end{array}$$

This shows that, if the fit is fully converged, fitting the spectral density or the correlation function is completely equivalent. However, the convergence behavior can obviously be different, as different aspects of the dynamics are weighted differently when fitting to one or the other quantity.

When fitting the correlation function, we minimize a weighted error,

(21) $$\mathrm{err}=\int_{0}^{t_{\max}}{\left|C\left(t\right)-C_{\mathrm{mod}}\left(t\right)\right|}^{2}\times \left|C\left(t\right)\right|dt,$$

where the local error is weighted by the absolute value of the correlation function so, when the correlation is stronger, the error has a more significant contribution. We found this definition to give better results than a “naive” fit without a weight, as it improves the fit in regions where the correlation function is larger and thus more important.

**Figure 6:**
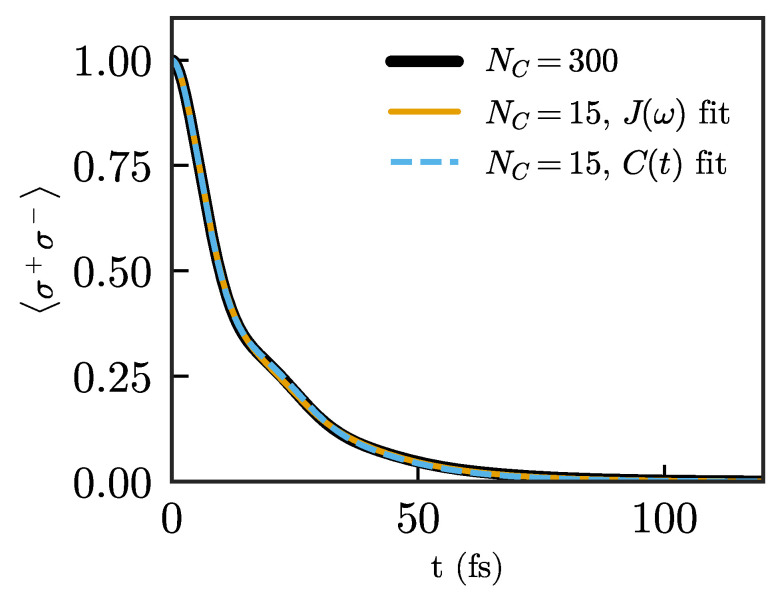
Population of the quantum emitter for $N_{C}=15$ after the fit to the spectral density (orange) and the fit to the correlation function (blue).

In both approaches, we let all chain parameters vary freely, using the parameters of the truncated chain as initial guesses for $\Omega_{i}$ and $\lambda_{i}$, and using small random numbers for the loss rates $\gamma_{i}$. Figure 6 shows the decay for the same quantum emitter for a chain of length $N_{C}=15$ after using both optimizations. We achieve comparably good descriptions of the emitter dynamics in both cases, using a much shorter chain compared to the previous approaches. While both methods offer similar results, we mention that we found the fit to the correlation function to be more stable and less sensitive to initial parameters than the fit to the spectral density.

### 3.2. Next-Nearest Neighbor Coupled Chain

In the previous section, we allowed the chain parameters to vary freely to obtain an optimized description of the bath spectral density, but maintained the topology of the chain with nearest-neighbor coupling, and only the first site coupled to the emitter. This restricts the possible freedom and implies that a chain with *N* sites has less flexibility to represent an arbitrary spectral density than a system of *N* modes with arbitrary couplings between each other and to the emitter, as used in [38]. In the current section, we demonstrate that relaxing these conditions to allow next-nearest neighbor coupling between the chain sites and the chain and emitter actually appears to be sufficient to represent an arbitrarily complex spectral density for a given number of sites *N*. To motivate this, we first summarize the model developed in Ref. [38], in which a collection of *N* mutually interacting discrete modes are all coupled to the emitter, leading to the model spectral density

(22) Jmod(ω)=1πg→·Im1H˜−ω·g→,

where $\tilde{H}$ is a complex symmetric N×N matrix with real offdiagonal terms, ${\tilde{H}}_{ij}=\omega_{ij}-i\delta_{ij}\gamma_{i}$, and $\vec{g}=\left(g_{1},g_{2},\ldots ,g_{N}\right)$ is a real coupling vector. We note that the expression given in Equation (18) is simply the specialization of this expression to the case where $\omega_{ij}$ is tridiagonal and $\vec{g}=\left(\lambda_{1},0,\ldots ,0\right)$. In the general form given here, the number of fit parameters is quite large, being equal to N(N+1)/2+N+N real numbers for $\omega_{ij}$, $\gamma_{i}$, and $g_{i}$, respectively. However, when $\tilde{H}$ is diagonalizable, $\tilde{H}=V\tilde{\Omega }V^{T}$, the same expression can be rewritten as

(23) Jmod(ω)=1πImG→·1Ω˜−ω·G→=1π∑iImGi2ω˜i−ω,

where $\vec{G}=\vec{g}V$ is the coupling vector transformed to the eigenbasis of $\tilde{H}$. In this form, the effective number of distinct parameters in the system is much smaller than in the original form, and is given by the 4N−1 real parameters necessary to describe the *N* complex eigenvalues ${\tilde{\omega }}_{i}$ and *N* complex coupling parameters $G_{i}$, with one less parameter due to the constraint that $\vec{G}\cdot \vec{G}=\vec{g}\cdot \vec{g}$ is real, i.e., Im∑iGi2=0. This shows that the original form, Equation (22), has many more parameters than necessary to characterize the spectral density. On the other hand, the tridiagonal chain form chosen in Equation (18) has only 3N free parameters (2N−1 for the real symmetric tridiagonal $\omega_{ij}$, *N* decay rates $\gamma_{i}$, one coupling $\lambda_{1}$), and thus necessarily induces hidden correlations in the values of $G_{i}$ and ${\tilde{\omega }}_{i}$ that determine the spectral density.

It is thus tempting to directly use the diagonal form Equation (23), in which the spectral density is written as a sum of simple poles (and the correlation function a sum of complex exponentials). However, in practice, this turns out to be difficult, as recently discussed in some detail in [25]. First, since $\vec{G}$ is complex, this form of the spectral density cannot be directly mapped to a physical system. Second, there are physical constraints that are not easily satisfied in this form. In particular, $J_{\mathrm{mod}}\left(\omega \right)$ has to be nonnegative for any ω, which is guaranteed when the loss rates $\gamma_{i}$ are all nonnegative in the original form [38], but not automatically fulfilled when fitting ${\tilde{\omega }}_{i}$ and $G_{i}$. Furthermore, even when such a set of parameters has been found, it is a highly nontrivial problem to find a complex orthogonal transformation matrix *V* to “undo” the diagonalization such that the resulting $g_{i}$ are real and $\tilde{H}$ can be mapped to a physical system, which furthermore requires that its imaginary part is positive definite. We are not aware of any constructive algorithm to achieve this, apart from the one developed for the case N=2 in Ref. [25]. It is thus advantageous to perform the fitting in the original form, where the physicality can be guaranteed by constraining all $\gamma_{i}$ to be nonnegative, and no additional work to find a physical system is necessary after the fit.

**Figure 7:**
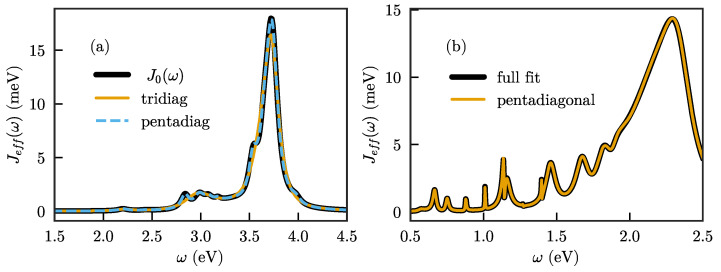
(**a**) Fit of the spectral density of Figure 2a when allowing next-nearest neighbor interactions in the chain, compared to a fit with only nearest neighbor interactions with the same number of modes (N=15); (**b**) fit of the spectral density of Ref. [38] using a chain with next-nearest neighbor interactions. Black line: Original fit using a full matrix $\omega_{ij}$, with all modes coupled to the emitter. Yellow line: Fit of the same data using a chain with only next-nearest neighbor interactions, and the emitter only coupled to the first two sites.

Given the above, an interesting question is whether the number of parameters in $\tilde{H}$ and $\vec{g}$ can be reduced while keeping the full flexibility of the method. It turns out that keeping to a chain form but allowing next-nearest neighbor interactions (also for the emitter, which thus interacts with the first two chain modes) gives exactly 4N−1 free parameters, equal to the number of actually independent degrees of freedom after diagonalization. The matrix $\tilde{H}$ is then pentadiagonal complex symmetric, with offdiagonal entries being real, while $\vec{g}=\left(g_{1},g_{2},0,\ldots ,0\right)$ has two (real) nonzero entries. This suggests that this form might be sufficient to obtain the optimal fit of any spectral density with a given number of modes *N*, while still maintaining the computationally advantageous property of a chain form with only short-range interactions between sites. In Figure 7a, we show that this form indeed permits a significantly improved description of the spectral density with the same number of modes.

While we are not aware of any formal proof that the chain with next-nearest neighbor interactions is indeed sufficient to represent any *N*-mode spectral density, we furthermore show the fit to the spectral density of the system treated in [38], which consists of a dual plasmonic nanoparticle antenna embedded inside a high-dielectric microsphere. The hybridization between whispering gallery modes of the microsphere and the plasmonic nanoparticle resonances leads to a complex spectral density with strong interference features [4,48]. As seen in Figure 7b, this complex spectral density is also well-represented when the fit is performed for a chain with next-nearest neighbor coupling. However, it should be mentioned here that, while the final fit does indeed reproduce the full spectral density, obtaining that fit is far from trivial. In particular, the fit with the next-nearest neighbor chain converges significantly worse and is much more sensitive to getting stuck in local minima than the fit with the full matrix. Obtaining a good fit for a complex spectral density then requires significant manual intervention (although it is possible that more advanced fitting methods than used here could mitigate this).

## 4. Discussion

We have discussed several approaches for truncating chain mappings of open quantum systems while maintaining an accurate description of the system dynamics. The approaches are based on adding dissipation to the chain modes in a way that minimizes reflections and thus reproduces the system dynamics optimally. As a first approach inspired by complex absorbing potentials, we used gradually increasing decay rates along the chain, which requires relatively long chains to work efficiently. We then showed that the necessary chain length can be reduced significantly by not using the formal chain mapping and then adding losses a posteriori, but by adjusting the chain parameters in a fitting procedure to reproduce either the environment spectral density or its correlation function. Finally, we found that relaxing the restriction on the chain form of the transformed system and permitting next-nearest neighbor coupling (also for the system itself) is enough to obtain the freedom to describe any spectral density characterized by $N_{C}$ resonances with a chain of $N_{C}$ sites, and adding more degrees of freedom (such as longer-range coupling along the chain) is not necessary for achieving the optimal description. Chains with next-nearest neighbor coupling could thus be seen as the “sweet spot” where the chain length is minimized and computational complexity is still limited (in particular, tensor network approaches can be expected to work well due to the quasi-1D nature of the problem).

## Data Availability

The data presented in this study are available upon request from the corresponding author.
